# Nut Consumption and Long-Term Risk of All-Cause Dementia: Preliminary Findings from Three Prospective Cohort Studies

**DOI:** 10.3390/nu18111722

**Published:** 2026-05-28

**Authors:** Mengjia Zhao, Minqing Yan, Leqi Fei, Hui Chen, Minyu Wu, Yuhui Li, Liyan Huang, Jie Shen, Gang Liu, Marta Guasch-Ferré, Jordi Salas-Salvadó, Changzheng Yuan

**Affiliations:** 1 Center of Clinical Big Data and Analytics of the Second Affiliated Hospital and School of Public Health, Zhejiang University School of Medicine, Hangzhou 310058, China; zhmj2521@zju.edu.cn (M.Z.); ymqthree@zju.edu.cn (M.Y.); 22318840@zju.edu.cn (L.F.); minyuwu@zju.edu.cn (M.W.); liyhui0910@163.com (Y.L.); jie.shen@zju.edu.cn (J.S.); 2 Zhejiang Key Laboratory of Intelligent Preventive Medicine, Hangzhou 310058, China; 3 Department of Neurology, The First Affiliated Hospital of Zhejiang University School of Medicine, Hangzhou 310003, China; hui.chen@zju.edu.cn; 4 Department of Clinical Pharmacy, The First Affiliated Hospital of Zhejiang University School of Medicine, Zhejiang University, Hangzhou 310003, China; huangliyan1997@zju.edu.cn; 5 Department of Nutrition and Food Hygiene, Hubei Key Laboratory of Food Nutrition and Safety, Ministry of Education Key Laboratory of Environment and Health, and State Key Laboratory of Environment Health (Incubating), School of Public Health, Tongji Medical College, Huazhong University of Science and Technology, Wuhan 430030, China; liugang026@hust.edu.cn; 6 Department of Nutrition, Harvard T.H. Chan School of Public Health, Boston, MA 02115, USA; marta.guasch@sund.ku.dk; 7 Department of Public Health, Faculty of Health and Medical Sciences, University of Copenhagen, 1353 Copenhagen, Denmark; 8 Novo Nordisk Foundation Center for Basic Metabolic Research, Faculty of Health and Medical Sciences, University of Copenhagen, 2200 Copenhagen, Denmark; 9 Alimentaciò, Nutrició, Desenvolupament i Salut Mental Grup (ANUT-DSM), Departament de Bioquímica i Biotecnologia, Universitat Rovira i Virgili, 43201 Reus, Spain; jordi.salas@urv.cat; 10 Centro de Investigación Biomédica en Red Fisiopatología de la Obesidad y la Nutrición, Institute of Health Carlos III, 28029 Madrid, Spain; 11 Institut d’Investigació Sanitària Pere Virgili, 43204 Reus, Spain

**Keywords:** nuts, diet, cognitive impairment, dementia, aging, prospective studies

## Abstract

**Background/Objectives:** Nuts have been associated with various health benefits, yet the evidence regarding their relationship with dementia is inconclusive. This study aims to examine the association between nut consumption and long-term risk of dementia across three prospective cohort studies. **Methods:** We analyzed data from adults aged 45 and older who were free of dementia at baseline in the Health and Retirement Study (HRS, 2013–2020), the Framingham Offspring Study (FOS, 1998–2018), and the Whitehall II Study (WHII, 2002–2016). Nut consumption, including tree nuts and peanuts, was assessed using validated food frequency questionnaires (FFQs) once at baseline in the HRS and repeatedly across multiple waves in the FOS and WHII. Incident all-cause dementia was identified through a validated algorithm in the HRS, expert panel reviews in the FOS, and healthcare record linkages in the WHII. Cox proportional hazards models were used to estimate the cohort-specific association between nut consumption and incident dementia, which were subsequently pooled. **Results:** Over 190,914 person-years of follow-up, 992 incident dementia cases occurred among 17,349 participants across the three cohorts. After multivariable adjustments, higher nut consumption was associated with a lower risk of dementia, with pooled hazard ratios (HRs) of 0.80 (95% confidence interval [CI]: 0.69–0.94; *I*^2^ = 0.0%) for 0.1–5.0 g/day vs. 0 g/day and 0.76 (95% CI: 0.58–0.99; *I*^2^ = 40.4%) for >5.0 g/day vs. 0 g/day (*p*-trend = 0.015). **Conclusions:** Our findings suggest that higher nut consumption was associated with a lower risk of incident dementia among middle-aged and older adults, with evidence of a dose-response trend observed in pooled analyses. These preliminary results support its inclusion as part of an overall brain-healthy dietary pattern.

## 1. Introduction

Dementia, characterized by the progressive impairment of cognitive domains and daily functioning [1], represents a mounting global health challenge as the population ages [2]. With effective treatments currently scarce, the emphasis on prevention strategies is imperative. Emerging evidence has raised interest in whether dietary modifications, particularly nut consumption, may offer neuroprotective benefits, although the overall evidence remains inconclusive [3,4].

The neuroprotective potential of nuts can be attributed to their distinct composition of bioactive compounds that target multiple pathological processes associated with dementia. Notably, walnuts are rich in α-linolenic acid (ALA), an omega-3 polyunsaturated fatty acid (PUFA) that is crucial for the integrity of neuronal membranes and the functioning of synapses [5,6,7]. Other types of nuts contribute to neuroprotection through different pathways, such as enhancing synaptic plasticity via magnesium, and offering antioxidant and anti-inflammatory benefits through vitamin E [8,9,10,11,12]. Furthermore, nuts may mitigate dementia risk through decreasing insulin resistance and beneficially affecting insulin action in brain regions involved in the modulation of metabolic and cognitive processes [13]. Additionally, nuts may also influence dementia risk indirectly through their well-established cardiovascular benefits. Nuts have been shown to improve blood lipid profiles, including reducing low-density lipoprotein cholesterol (LDL-C), and to lower the risk of cardiovascular disease [14]. This is particularly relevant given that the 2024 Lancet Commission on dementia prevention, intervention, and care identified LDL-C as a modifiable risk factor for dementia, and given the substantial overlap between cardiovascular disease and dementia, especially vascular dementia [15]. Thus, the cardioprotective effects of nuts may represent an important mechanistic pathway linking nut consumption to reduced dementia risk.

In population-based epidemiological studies, nuts have been associated with various health benefits, including lower risk of cardiovascular disease, diabetes, and cancers [13,16]. However, the evidence regarding their relationship with dementia is inconclusive. Prospective cohort analyses suggested potential cognitive benefits, though findings vary substantially across studies [17]. For instance, in a prospective study involving 50,386 participants from the UK Biobank (UKB) with a mean follow-up of 7.1 years, a 12% lower risk of all-cause dementia was reported among daily nut consumers compared to non-consumers [18]. In contrast, the Three-City (3C) Bordeaux study, involving 1,412 French older adults, found no significant association between nut consumption and dementia risk after an average follow-up of 9.7 years [19]. These discrepancies may be related to differences in study populations, sample size, and confounding adjustment, as well as the inherent limitation of residual confounding in observational studies, where nut consumption may act as a marker of overall health-related behaviors and lifestyle patterns. Randomized clinical trials (RCTs) examining the effects of nut consumption on cognitive outcomes are few, and results have been contradictory, showing either beneficial or no effects on cognitive function. In the PREDIMED (PREvención con DIeta MEDiterránea) trial, a multicenter randomized controlled trial conducted in Spain, participants assigned to a Mediterranean diet supplemented with either nuts or extra-virgin olive oil (EVOO) showed better cognitive performance compared to those on a low-fat control diet over a 6.5-year follow-up period; however, the beneficial effects were more pronounced in the EVOO-supplemented group [20]. Several other studies reported null associations [21,22]. Overall, the available population-based evidence is insufficient to establish definitive recommendations.

Moreover, nuts are prominently featured in general dietary guidelines, but specific recommendations vary by dietary pattern and region. The Mediterranean-Dietary Approaches to Stop Hypertension (DASH) Intervention for Neurodegenerative Delay (MIND) diet advises consuming ≥5 servings weekly for neuroprotection [23], while cardiometabolic-focused guidelines recommended 30 g/day in the Mediterranean diet and 4–5 servings/week for the Dietary Approaches to Stop Hypertension (DASH) diets [24,25]. These recommendations are mirrored in national dietary guidelines, with the US suggesting ≥5 ounces/week and the UK recommending 30 g/day [26,27]. Additionally, data from the Global Dietary Database (GDD) indicates that the population average of daily nut consumption was only 5 g [28], which accounts for just 17–25% of the minimum targets set by nut consumption recommendations. This substantial gap between guidelines and actual population intake underscores the need for high-quality studies to quantify the dose-response relationship across the full range of intake levels, particularly at lower doses that represent achievable public health targets. Prospective cohort studies with large sample sizes and detailed dietary assessments are well-suited to address this question by providing precise estimates of association across diverse populations.

Our objective was to investigate the association between nut consumption and the long-term risk of dementia using data from three prospective cohort studies: the Health and Retirement Study (HRS), the Framingham Offspring Study (FOS), and the Whitehall II Study (WHII). By leveraging these diverse cohorts, we sought to provide more robust evidence and explore potential dose-response relationships.

## 2. Materials and Methods

### 2.1. Study Population

We utilized individual-level data from three prospective studies. The HRS is a nationally representative cohort study of over 37,000 adults aged 50 and above in the USA since 1992 [29]. Within this framework, 8035 participants completed a dietary assessment through the 2013 Health Care and Nutrition Study (HCNS) [30], followed by cognitive assessments in 2014, 2016, 2018, and 2020. The Framingham Heart Study, a community-based cohort study initiated in 1948, expanded in 1971 with the formation of the FOS cohort, including the children of the original cohort and their spouses [31]. FOS participants underwent dietary assessments during examinations 5 (1991–1995), 6 (1995–1998), 7 (1998–2001), and 8 (2005–2008), with ongoing dementia monitoring until 2018 [32]. The WHII was established in 1985–1988 among 10,308 civil service workers aged 35–55, with follow-up clinical examinations conducted approximately every four to five years since baseline [33]. All participants provided written informed consent prior to data collection. The HRS was approved by the University of Michigan Institutional Review Board (IRB), the FOS by the Boston University Medical Center IRB, and the WHII by the University College London Medical School Committee.

**Figure 1:**
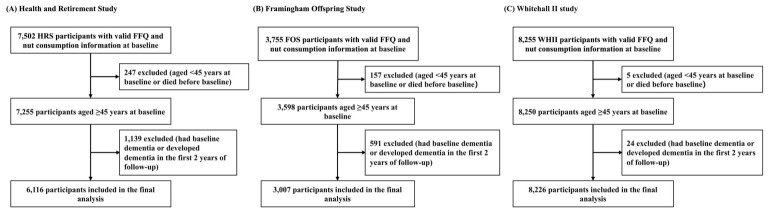
Flowcharts of participant inclusion in the cohort analyses.

In the present study, we conducted a longitudinal follow-up of participants enrolled in the HRS from baseline (2013) to 2020, in the FOS from baseline (examination 7, 1998–2001) to 2018, and the WHII from baseline (phase 7, 2002–2004) to 2016. In our study, we included individuals aged 45 years and older who completed one or more valid food frequency questionnaires (FFQs) with a total energy intake range of 500–4500 kcal [34]. Participants who exhibited dementia at baseline or developed dementia within the first 2 years of follow-up were excluded (Figure 1A–C).

### 2.2. Dietary Assessment

Dietary intake was assessed using a validated semi-quantitative FFQ [35], with cohort-specific modifications across the three studies. For nut consumption, participants were asked about their average frequency of consuming a standard serving of nuts (defined as 28.35 g or 1 ounce) over the preceding year. To balance exposure distribution and statistical stability, and to adopt a commonly used threshold in nutritional epidemiology for nut-related health studies [36], nut consumption was categorized into 0, 0.1–5.0, and >5.0 g/day groups for all three cohorts. Specifically, the FOS (examinations 5–7, 1991–2001) and WHII (phases 3/5/7, 1991–2003) calculated average nut consumption from repeated assessments, while the HRS utilized a single baseline assessment (2013). In the HRS, additional analyses were performed using quintiles of nut consumption and the recommended nut consumption threshold (<5, ≥5 ounces/week) [27].

### 2.3. Ascertainment of Incident Dementia

The three cohort studies employed distinct strategies for identifying incident dementia cases, with all utilizing all-cause dementia definitions rather than subtype-specific classifications due to methodological differences in ascertainment approaches. The HRS relied on validated cognitive testing algorithms [37], the FOS incorporated multidisciplinary expert adjudication [38], and the WHII utilized comprehensive electronic health record linkages [39] (see Text S1 for detailed diagnostic criteria and subtype considerations).

### 2.4. Assessment of Covariates

The following potential confounders were included in the analysis: age, sex (male, female), race (White/Caucasian, others), marital status (married, others), education level (high school or below, college or above), income level (tertiles: low, middle, or high), body mass index (BMI) (<25 kg/m^2^, 25.0–<30.0 kg/m^2^, or ≥30.0 kg/m^2^), smoking status (current, non-current), physical activity (low, high), hypertension (yes, no), diabetes (yes, no), heart disease (yes, no), stroke (yes, no), depressive symptoms (yes, no), total energy intake, and the modified MIND diet score, which was adapted from our prior methodology and calculated excluding the nut component to avoid overadjustment [40]. This score served as a comprehensive indicator of overall dietary quality, as the remaining 14 components capture a wider range of dietary patterns than adjusting for only a few individual food groups, thus providing more comprehensive control for confounding from other dietary factors. Detailed calculation methods, including cohort-specific adjustments, were provided in Text S2. Alcohol intake, specifically wine, was a component of the MIND diet score and was therefore incorporated into the multivariable models through the modified MIND diet score rather than as an independent covariate. Missing covariate data were handled using multiple imputation by chained equations (MICE) with 20 imputed datasets, incorporating all covariate variables to satisfy the missing-at-random assumption (missing data characteristics presented in Table S1). Missingness was generally low (<5%) for most variables, with the exception of a few covariates, such as income level in the FOS (28.1%) and depressive symptoms in the WHII study (30.5%).

### 2.5. Statistical Analysis

Baseline characteristics of participants were presented as counts (percentages) for categorical variables and means (standard deviations [SDs]) for continuous variables in each cohort. After confirming the proportional hazards assumptions were met, we used Cox proportional hazards models with follow-up time as the underlying time scale to assess the association between nut consumption and incident dementia in the primary analysis. This approach allowed us to account for the non-linear relationship between age and dementia risk while using the full follow-up period. Hazard ratios (HRs) and 95% confidence intervals (CIs) were estimated using three multivariable models. Model 1 was adjusted for age, age square, sex, race, marital status, education level, income level, BMI, smoking status, and physical activity. Model 2 was adjusted for variables in Model 1 plus hypertension, diabetes, heart disease, stroke, depressive symptoms, and total energy intake. Model 3 was adjusted for the MIND diet score (excluding nut consumption) in addition to the variables in Model 2. The *p* value for trend was calculated using the Wald test by treating nut consumption as a continuous variable, where each category was assigned the median number of servings of nuts consumed per day. All analyses were performed independently within each cohort, and the HRs and 95% CIs from all three models were subsequently pooled across the three cohorts using random-effects models to generate comprehensive summary estimates while allowing for between-study heterogeneity. Additionally, we further explored the association between quintiles of nut consumption and incident dementia, along with the association between recommended nut consumption and incident dementia in the HRS. These analyses were not conducted in the FOS and WHII cohorts because preliminary data screening revealed insufficient variability in nut consumption patterns and inadequate numbers of dementia cases in higher intake categories to ensure statistical reliability.

In the secondary analysis, we performed subgroup analyses by age (<65 years, ≥65 years), sex (male, female), education level (high school or below, college or above), income level (low, medium/high), BMI (<25.0 kg/m^2^, ≥25.0 kg/m^2^), current smoking status (yes, no), and physical activity (low, high). We tested for potential interactions using likelihood ratio tests.

We conducted several sensitivity analyses to assess the robustness of our findings. First, we performed complete-case analyses, excluding participants with any missing covariate data, to evaluate the robustness of the multiple imputation approach. Second, we restricted analyses to participants without a history of stroke at baseline to minimize confounding effects on the nut consumption-dementia association. Third, we excluded participants who developed dementia within the first five years of follow-up to mitigate reverse causality. Fourth, we refined our multivariable models by adjusting for individual components of the MIND diet rather than the overall score. Finally, we additionally adjusted for current alcohol consumption (yes, no), given its potential dietary and behavioral correlations with nut consumption.

Analyses were performed using R version 4.3.1. Statistical tests were two-sided, and *p* values < 0.05 were considered statistically significant.

## 3. Results

### 3.1. Baseline Characteristics

In the cohort analyses, we included 6116 participants from the HRS (mean [SD] age, 65.8 [10.0] years; 59.3% female), 3007 participants from the FOS (mean [SD] age, 61.2 [8.7] years; 54.5% female), and 8226 participants from the WHII (mean [SD] age, 61.4 [6.0] years; 31.0% female) (Table 1). The mean (SD) baseline nut consumption was 8.7 (15.1) g/day in the HRS, 3.0 (5.7) g/day in the FOS, and 4.1 (7.8) g/day in the WHII.

### 3.2. Outcome Analysis

Over 190,914 person-years, 992 incident dementia cases were identified among 17,349 participants with normal cognition at baseline (532 in the HRS, 262 in the FOS, and 198 in the WHII; Table 2 and Table S2). After multivariable adjustment, higher nut consumption (>5.0 g/day in the HRS and 0.1–5.0 g/day in the WHII) was associated with a lower risk of dementia compared to non-consumers (HR = 0.61, 95% CI: 0.46–0.81 in the HRS; HR = 0.72, 95% CI: 0.52–0.99 in the WHII). Although not statistically significant, FOS results exhibited comparable inverse trends (HR = 0.87, 95% CI: 0.65–1.15 for 0.1–5.0 g/day and HR = 0.90, 95% CI: 0.60–1.34 for >5.0 g/day). Pooled analyses showed inverse associations for both 0.1–5.0 g/day (HR = 0.80, 95% CI: 0.69–0.94; *I*^2^ = 0.0%) and >5.0 g/day (HR = 0.76, 95% CI: 0.58–0.99; *I*^2^ = 40.4%) versus non-consumers (*p*-trend = 0.015).

In the HRS, higher nut consumption was associated with a lower risk of incident dementia, with HRs showing a generally inverse pattern across increasing intake levels (*p*-trend < 0.001; Table 3). In fully adjusted models, a significantly lower risk was first observed at the third quintile (Q3: HR = 0.67, 95% CI: 0.52–0.87), remained significant at Q4 (HR = 0.75, 95% CI: 0.58–0.97), and was most pronounced at Q5 (HR = 0.53, 95% CI: 0.39–0.72). No significant association was found for Q2 (HR = 0.93, 95% CI: 0.73–1.19). Although the pattern was not strictly monotonic, with a larger reduction at Q3 followed by a modest attenuation at Q4, the overall trend suggested that higher intake levels were generally associated with lower dementia risk, with the strongest reduction observed at the highest intake level. Further analyses incorporating an alternative exposure definition yielded consistent results, showing that meeting nut consumption recommendations was associated with a 30% lower dementia risk compared to non-adherence (HR = 0.70, 95% CI: 0.51–0.94; *p*-trend = 0.019; Table S3).

### 3.3. Subgroup Analysis

**Figure 2:**
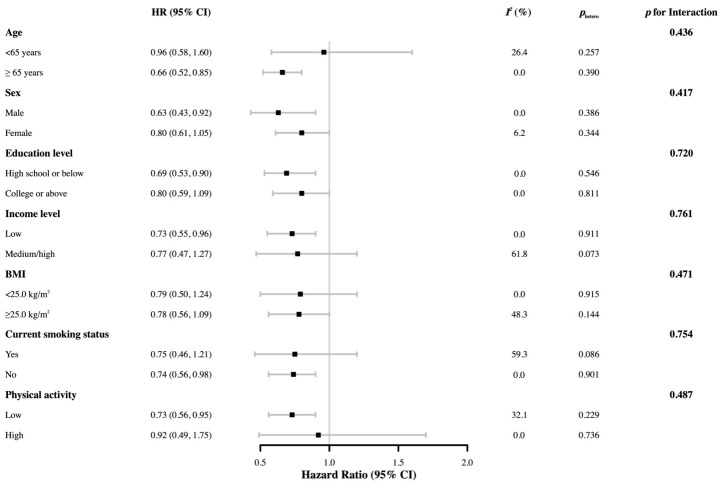
Pooled subgroup analysis of nut consumption (>5.0 g/day vs. 0 g/day) and incident dementia. Cox proportional hazards model adjusted for age, age square, sex, race, marital status, education level, income level, BMI, smoking status, physical activity, hypertension (yes or no), diabetes, heart disease, stroke, depressive symptoms, total energy intake, and the modified MIND diet score. Study estimates from three cohorts were pooled using a random-effects model.

Pooled analyses across predefined subgroups indicated a generally consistent association between higher nut consumption (>5 g/day) and lower dementia risk, with no statistically significant effect modification observed (all *p*-interaction > 0.4; Figure 2). The inverse association appeared more pronounced among participants aged ≥65 years (HR = 0.66, 95% CI: 0.52–0.85), males (HR = 0.63, 95% CI: 0.43–0.92), and those with a high school education or below (HR = 0.69, 95% CI: 0.53–0.90), although the differences across strata were not statistically significant. Similar associations were found across categories of income, BMI, smoking status, and physical activity, despite some variation in point estimates. *I*^2^ values indicated generally low to moderate heterogeneity between subgroups, suggesting that the inverse association between nut consumption and dementia risk was broadly consistent across demographic and lifestyle subgroups. Detailed cohort-specific subgroup results are presented in Table S4, with no significant interactions detected in any cohort.

### 3.4. Sensitivity Analysis

Sensitivity analyses supported the robustness of the main findings (Table S5). Complete-case analyses excluding participants with any missing covariate data yielded results directionally consistent with the primary imputed analyses. Compared with non-consumers, the pooled hazard ratio for 0.1–5.0 g/day was 0.74 (95% CI: 0.60–0.90; *I*^2^ = 0.0%) and for >5.0 g/day was 0.82 (95% CI: 0.52–1.28; *I*^2^ = 59.1%). When excluding participants with stroke at baseline, the inverse association between higher nut consumption and dementia remained statistically significant. Compared with non-consumers, the pooled HRs were 0.82 (95% CI: 0.69–0.97; *I*^2^ = 0.0%) for 0.1–5.0 g/day and 0.71 (95% CI: 0.56–0.90; *I*^2^ = 13.4%) for >5.0 g/day (*p*-trend = 0.004). Additional adjustments for individual components of the MIND diet rather than the overall score yielded consistent associations: pooled HRs of 0.85 (95% CI: 0.72–1.00; *I*^2^ = 0.0%) and 0.80 (95% CI: 0.64–1.00; *I*^2^ = 5.4%) for 0.1–5.0 g/day and >5.0 g/day, respectively (*p*-trend = 0.021). Similar associations were observed after adjusting for current drinking status (pooled HR = 0.80, 95% CI: 0.69–0.94; *I*^2^ = 0.0% for 0.1–5.0 g/day; pooled HR = 0.75, 95% CI: 0.58–0.96; *I*^2^ = 29.2% for >5.0 g/day; *p*-trend = 0.016). However, excluding dementia cases within the first 5 years attenuated the associations, with pooled HRs of 0.83 (95% CI: 0.70–0.99; *I*^2^ = 0.0%) for 0.1–5.0 g/day and 0.79 (95% CI: 0.56–1.10; *I*^2^ = 57.4%) for >5.0 g/day, suggesting potential reverse causation or reduced statistical power.

## 4. Discussion

We integrated data from three large prospective cohorts, including the HRS, FOS, and WHII, to assess the association between nut consumption and long-term risk of dementia among 17,349 middle-aged and older adults without dementia at baseline. Over follow-up periods extending to 18 years, multivariable-adjusted models demonstrated a significant inverse association between nut consumption and incident dementia. The pooled analyses revealed that consuming 0.1–5.0 g/day of nuts was associated with a 20% lower dementia risk (HR = 0.80, 95% CI: 0.69–0.94), while intake exceeding 5.0 g/day showed a 24% lower risk (HR = 0.76, 95% CI: 0.58–0.99) compared to non-consumers, with a statistically significant dose-response trend (*p*-trend = 0.015). However, this pattern was not fully consistent across individual cohorts, particularly for the highest intake category, and should therefore be considered preliminary and interpreted with caution. Consistently, a quintile-based analysis in the HRS showed a clear graded association, with an overall decreasing trend in dementia risk from the lowest to the highest nut consumption group (*p*-trend < 0.001), despite minor fluctuations across intermediate quintiles.

These findings align with and build on previous epidemiological evidence linking nut consumption to reduced dementia risk. For instance, a prospective study in the UKB, including 50,386 participants over 7.1 years of follow-up, reported a 12% reduction in all-cause dementia risk among daily nut consumers (HR = 0.88, 95% CI: 0.83–0.94) [18], compared to non-consumers. The point estimate in our pooled analysis for intakes above 5.0 g/day (HR = 0.76, 95% CI: 0.58–0.99) is somewhat lower than the UKB estimate, but the confidence intervals from the two studies overlap substantially (UKB: 0.83–0.94; our study: 0.58–0.99). The narrower confidence interval in the UKB reflects its larger sample size and correspondingly greater precision. Taken together, the two studies are broadly compatible, providing consistent evidence of an inverse association between nut consumption and dementia risk, with our study offering additional detail on the dose-response relationship across different intake levels. Notably, participants in the HRS cohort who met the US dietary guideline recommendation (≥5 ounces/week, approximately 20.25 g/day) [27] exhibited a significantly lower risk of dementia (HR = 0.70, 95% CI: 0.51–0.94), which may support current dietary recommendations. Moreover, our results also suggest that even a relatively low level of nut consumption (0.1–5.0 g/day), substantially below current dietary recommendations, was associated with a statistically significant reduction in dementia risk, highlighting the potential benefits of small dietary improvements even in low-consumption populations.

While the French Three-City study reported a hazard ratio of 0.92 (95% CI: 0.76–1.12) for nut consumption [19], its confidence interval overlaps with our pooled estimate and is compatible with a reduction in dementia risk. The wider confidence interval in that study reflects its smaller sample size and lower median nut consumption (1.2 g/day). Similarly, in our FOS cohort, the estimates for 0.1–5.0 g/day (HR = 0.87, 95% CI: 0.65–1.15) and >5.0 g/day (HR = 0.90, 95% CI: 0.60–1.34) are directionally consistent with the overall pattern, albeit with wider confidence intervals due to lower statistical power and exposure variability. Despite this, the pooled analysis across all three cohorts still showed a significant dose-response trend, supporting the overall inverse association.

Sensitivity analyses reinforced the robustness of the main findings. Excluding participants with stroke at baseline, adjusting for individual MIND diet components, or controlling for current drinking status all yielded generally consistent results with the primary analysis. However, when excluding dementia cases within the first 5 years of follow-up, the association for intakes above 5.0 g/day was attenuated, with a widened confidence interval. While this loss of precision is largely attributable to reduced sample size and statistical power, we cannot entirely exclude the possibility of reverse causation, whereby early, undiagnosed dementia may have influenced dietary habits prior to baseline. It is possible that individuals with prodromal dementia might reduce their nut consumption due to changes in appetite, food preferences, or ability to prepare or consume certain foods, which could contribute to the observed attenuation.

Several biologically plausible mechanisms may explain our findings. Nuts contain a rich array of bioactive components, including PUFAs, essential vitamins (e.g., vitamin E and folate), and various minerals (e.g., magnesium and selenium) that collectively support brain health through complementary pathways. For instance, PUFAs, particularly omega-3 fatty acids, are integral to neuronal membrane integrity and synaptic plasticity [7], while vitamin E and phenolic compounds exhibit potent antioxidant capacity along with notable anti-inflammatory properties that mitigate oxidative stress and pro-inflammatory cytokine production [8,9,10,11,12]. These components may collectively modulate critical pathways such as nuclear factor kappa B (NF-κB), proliferator-activated receptor γ (PPAR-γ), and brain-derived neurotrophic factor (BDNF) signaling, and possibly inhibit β-secretase-mediated amyloidogenesis [41,42,43,44]. Additionally, nuts may also confer cerebrovascular benefits by improving blood lipid profiles, endothelial function, and insulin sensitivity [13,45,46,47,48,49,50]. Emerging evidence further points to potential gut microbiota modulation as an additional neuroprotective pathway, suggesting the multidimensional mechanisms through which nuts may influence brain health [51]. However, these mechanistic pathways have not been directly tested in our study, and further research is needed to establish causality and elucidate underlying mechanisms.

The strengths of our study include its large sample size, prospective design, repeated dietary and cognitive assessments, and rigorous adjustment for potential confounders. However, several limitations should be acknowledged. First, nut consumption was assessed using food frequency questionnaires, which are susceptible to recall error and misreporting. Second, despite our comprehensive adjustments for a wide range of sociodemographic, lifestyle, and health-related factors, residual confounding remains possible. Individuals with higher nut consumption may also engage in other health-promoting behaviors, including greater health awareness, higher adherence to other healthy dietary patterns, and more frequent physical activity, some of which we could not fully measure or adjust for. Therefore, the observed inverse association may partly reflect broader healthy lifestyle patterns rather than an independent effect of nut consumption. Third, the observational study design inherently limits causal inference, underscoring the need for RCTs to establish definitive evidence of causality. Fourth, the three cohorts employed different methods for dementia ascertainment: a validated cognitive testing algorithm in the HRS, multidisciplinary expert adjudication in the FOS, and electronic health record linkage in the WHII. While each method was validated within its respective study context, these differences may introduce heterogeneity in outcome classification. For instance, health record linkage may underestimate dementia incidence compared with active surveillance, whereas cognitive algorithms may have different sensitivity and specificity profiles across populations. This methodological heterogeneity may have contributed to the observed variation in cohort-specific effect estimates. Importantly, our outcome definition encompassed all-cause dementia rather than specific subtypes (e.g., Alzheimer’s disease, vascular dementia), which may obscure potential differential relationships given their distinct etiopathologies. Additionally, our assessment of nut consumption did not distinguish between specific nut subtypes or processing methods (e.g., raw, roasted, or salted). Different nuts vary in their nutritional composition and may exert neuroprotective effects through different biological mechanisms. Future studies using more detailed nut-specific dietary assessment are needed to clarify these potential differences. Finally, the study focused on Western participants, which may limit the generalizability of the findings to other populations.

## 5. Conclusions

Our findings suggest an association between higher nut consumption and a lower risk of incident dementia, with a dose-response trend observed in pooled analyses. These preliminary results support the inclusion of nuts as part of an overall brain-healthy dietary pattern. However, given the variability across cohorts, these findings should be interpreted cautiously. Further research is needed to address the limitations of the current study, explore the specific mechanisms through which nuts may exert their protective effects, and identify the optimal dose of nuts to maximize their potential benefits for brain health.

## Figures and Tables

**Table 1:** Baseline characteristics of participants in the Health and Retirement Study, the Framingham Offspring Study, and the Whitehall II study *.

Variables	Health and Retirement Study (N = 6116)	Framingham Offspring Study (N = 3007)	Whitehall II Study (N = 8226)
Age, mean (SD), y	65.8 (10.0)	61.2 (8.7)	61.4 (6.0)
Female, No. (%)	3625 (59.3)	1639 (54.5)	2554 (31.0)
White/Caucasian, No. (%)	4695 (76.8)	2646 (88.0)	7490 (91.1)
Married, No. (%)	3900 (63.8)	2508 (83.4)	5849 (71.1)
Education level, No. (%)			
High school or below	2941 (48.1)	1152 (38.3)	2451 (29.8)
College or above	3175 (51.9)	1855 (61.7)	5775 (70.2)
Income level, No. (%)			
Low	1580 (25.8)	1248 (41.5)	3943 (47.9)
Medium	2136 (34.9)	929 (30.9)	1594 (19.4)
High	2400 (39.2)	830 (27.6)	2689 (32.7)
Current smoker, No. (%)	719 (11.8)	382 (12.7)	865 (10.5)
Physical activity, No. (%)			
Low	4356 (71.2)	1778 (59.1)	6887 (83.7)
High	1760 (28.8)	1229 (40.9)	1339 (16.3)
Body mass index, No. (%), kg/m^2^			
<25.0	1595 (26.1)	874 (29.1)	3194 (38.8)
25.0–<30.0	2265 (37.0)	1252 (41.6)	3585 (43.6)
≥30.0	2256 (36.9)	881 (29.3)	1447 (17.6)
Total energy intake, mean (SD), kcal/d	1783.8 (739.5)	1855.6 (547.2)	1994.6 (589.6)
Nut consumption, mean (SD), g/d	8.7 (15.1)	3.0 (5.7)	4.1 (7.8)
Hypertension, No. (%)	3425 (56.0)	1941 (64.5)	4819 (58.6)
Diabetes, No. (%)	1234 (20.2)	353 (11.7)	1614 (19.6)
Heart disease, No. (%)	1228 (20.1)	375 (12.5)	2791 (33.9)
Stroke, No. (%)	348 (5.7)	50 (1.7)	253 (3.1)
Depressive symptoms, No. (%)	702 (11.5)	466 (15.5)	1324 (16.1)
Follow-up duration, mean (SD), y	7.3 (1.4)	13.7 (4.8)	12.8 (1.0)

* Variable definitions differed across cohorts as follows: household income was used in the HRS and WHII, whereas personal income was assessed in the FOS; physical activity was measured as the frequency of vigorous activity (days per week) in the HRS, weekly bouts of intense activity in the FOS, and the duration of vigorous activity (hours per week) in the WHII; depressive symptoms were assessed using the 8-item Center for Epidemiologic Studies-Depression (CES-D) scale in the HRS, 20-item CES-D scale in the FOS, and clinical records linkage in the WHII.

**Table 2:** Multivariable-adjusted Hazard Ratios (HRs) and 95% CIs of nut consumption and incident dementia *.

Variables	Nut Consumption			
	0 g/Day	0.1–5.0 g/Day	>5.0 g/Day	*p* Value for Trend
Health and Retirement Study				
Cases/Person-years	91/4480	304/24,216	137/16,000	
Nut consumption, median (Q1, Q3)	0	1.42 (0.95, 2.84)	14.51 (7.83, 26.80)	
HR (95% CI)	1 (Reference)	0.81 (0.64, 1.03)	0.61 (0.46, 0.81)	0.001
Framingham Offspring Study				
Cases/Person-years	89/10,643	132/23,577	41/6943	
Nut consumption, median (Q1, Q3)	0	1.89 (0.95, 2.61)	8.75 (6.08, 12.15)	
HR (95% CI)	1 (Reference)	0.87 (0.65, 1.15)	0.90 (0.60, 1.34)	0.718
Whitehall II study				
Cases/Person-years	74/27,956	81/54,064	43/23,035	
Nut consumption, median (Q1, Q3)	0	2.02 (1.35, 2.70)	10.10 (7.09, 14.20)	
HR (95% CI)	1 (Reference)	0.72 (0.52, 0.99)	0.87 (0.59, 1.29)	0.866
Pooled ^†^				
HR (95% CI)	1 (Reference)	0.80 (0.69, 0.94)	0.76 (0.58, 0.99)	0.015
*I*^2^ (%)		0.0	40.4	0.0
*p*_hetero_		0.688	0.187	0.622

* Cox proportional hazards model was adjusted for age, age square, sex, race, marital status, education level, income level, BMI, smoking status, physical activity, hypertension, diabetes, heart disease, stroke, depressive symptoms, total energy intake, and the modified MIND diet score. ^†^ Study estimates from three cohorts were pooled using a random-effects model.

**Table 3:** Association between nut consumption (categorized into quintiles) and incident dementia in the Health and Retirement Study.

Variables	Quintile 1	Quintile 2	Quintile 3	Quintile 4	Quintile 5	*p* Value for Trend
Cases/Person-years	190/11,074	97/6944	94/9472	89/8418	62/8788	
Nut consumption, median (Q1, Q3)	0.47 (0.00, 0.95)	1.42 (1.42, 1.42)	2.84 (2.84, 4.25)	6.41 (5.67, 9.99)	26.20 (18.10, 36.40)	
Model 1, HR (95% CI) *	1 (Reference)	0.91 (0.71, 1.16)	0.68 (0.53, 0.87)	0.78 (0.61, 1.01)	0.56 (0.42, 0.76)	0.001
Model 2, HR (95% CI) ^†^	1 (Reference)	0.93 (0.73, 1.19)	0.67 (0.52, 0.86)	0.74 (0.57, 0.96)	0.52 (0.38, 0.70)	<0.001
Model 3, HR (95% CI) ^‡^	1 (Reference)	0.93 (0.73, 1.19)	0.67 (0.52, 0.87)	0.75 (0.58, 0.97)	0.53 (0.39, 0.72)	<0.001

* Model 1 was adjusted for age, age square, sex, race, marital status, education level, income level, BMI, smoking status, and physical activity. ^†^ Model 2 was adjusted for variables in Model 1 plus hypertension, heart disease, stroke, depressive symptoms, and total energy intake. ^‡^ Model 3 was adjusted for variables in Model 2 plus the modified MIND diet score.

## Data Availability

The Health and Retirement Study data are publicly available from the HRS website (https://hrs.isr.umich.edu/, accessed on 30 November 2024). Data from the Framingham Heart Study can be accessed by submitting a research proposal through the study group (https://www.framinghamheartstudy.org/, accessed on 30 November 2024) upon reasonable request. Data from the Whitehall II Study can be accessed upon reasonable request on the Dementias Platform UK (https://www.dementiasplatform.uk/, accessed on 30 November 2024).

## Supplementary Materials

The following supporting information can be downloaded at https://www.mdpi.com/article/10.3390/nu18111722/s1. Text S1: Dementia ascertainment criteria and subtype classification considerations for detailed description; Text S2: Calculation of the modified Mediterranean-DASH Intervention for Neurodegenerative Delay (MIND) Diet score; Table S1: No. (%) of missing data across the three cohorts; Table S2: Association between nut consumption and incident dementia: results from Model 1 and Model 2; Table S3: Association between recommended nut consumption and incident dementia in the HRS; Table S4: Subgroup analyses of nut consumption (>5.0 g/day vs. 0 g/day) and incident dementia in the three cohorts; Table S5: Association between nut consumption and incident dementia in sensitivity analyses.
