# Supplementary material for: Nut Consumption and Long-Term Risk of All-Cause Dementia: Preliminary Findings from Three Prospective Cohort Studies
Source: Nutrients. 2026 May 28;18(11):1722. doi: 10.3390/nu18111722 (PMC13258416; doi:10.3390/nu18111722)
Supplement: Supplementary file 1 [file nutrients-18-01722-s001.zip › nutrients-4304267-supplementary.pdf]

Text S1. Dementia ascertainment criteria and subtype classification considerations for detailed description

The three cohort studies employed distinct but rigorously validated strategies for identifying incident dementia cases, each with varying capacity for subtype classification. The Health and Retirement Study (HRS) utilized the Langa-Weir Classification algorithm, which defines dementia through standardized cognitive assessment thresholds: a score ≤ 6 on a 27-point cognitive battery for self-respondents or ≥ 6 on an 11-point proxy questionnaire, providing reliable case identification while not distinguishing specific dementia subtypes. The Framingham Offspring Study (FOS) employed a comprehensive adjudication process conducted by neurologist-neuropsychologist panels. This approach incorporated serial neuropsychological testing, detailed medical record review, and available neuropathological data to establish both dementia diagnosis and subtype classification. The reliability of subtype determinations in FOS was optimized through systematic integration of longitudinal clinical evidence across examination cycles, though dependent on documentation completeness over time. The Whitehall II study (WHII) ascertained dementia cases through linked electronic health records, which permitted broad categorization by International Classification of Diseases (ICD) codes but lacked the granularity to reliably classify less common or mixed dementia subtypes. These inherent differences in subtype ascertainment methodology across cohorts necessitated the use of all-cause dementia as the primary analytic endpoint. This approach mitigated potential misclassification bias arising from heterogeneous subtype diagnostic criteria while maintaining statistical power for cross-cohort comparisons.

Text S2. Calculation of the modified Mediterranean-DASH Intervention for Neurodegenerative Delay (MIND) Diet score

The Mediterranean-DASH Intervention for Neurodegenerative Delay (MIND) diet score was calculated based on 15 dietary components (10 beneficial and 5 restricted groups). For olive oil specifically, participants received a score of 1 if they reported using it as their primary cooking oil and 0 otherwise. Other beneficial components (e.g., green leafy vegetables, berries) were scored 0–1 by intake tertiles, while restricted components (e.g., red meats, fried foods) were inversely scored. Study-specific modifications included: (1) exclusion of nuts (primary exposure) from the total score (reducing maximum to 14), and (2) omission of olive oil scoring in WHII due to data unavailability (maximum 13). All other components maintained original MIND diet algorithms, including adapted wine scoring (1 glass/day=1; 1/mo-6/wk=0.5; 0/>1 glasses/day=0). The final cohort-specific scores thus ranged 0–14 (HRS/FOS) and 0–13 (WHII), with higher values indicating better adherence.

Table S1. No. (%) of missing data across the three cohorts

Variables	HRS	FOS	WHII
Ethnicity	15 (0.2)	-	29 (0.4)
Education level	30 (0.5)	433 (14.4)	1543 (18.8)
Marital status	3 (0.0)	429 (14.3)	27 (0.3)
Income level	-	846 (28.1)	1293 (15.7)
Smoking status	29 (0.5)	-	155 (1.9)
Physical activity	16 (0.3)	68 (2.3)	2014 (24.5)
Body mass index	59 (1.0)	1 (0.0)	166 (2.0)
Hypertension	-	-	900 (10.9)
Diabetes	-	6 (0.2)	292 (3.5)
Heart disease	-	734 (24.4)	694 (8.4)
Stroke	-	226 (7.5)	1947 (23.7)
Depressive symptoms	111 (1.8)	628 (20.9)	2511 (30.5)

Values are presented as No. (%). Percentages were calculated based on the total sample size of each cohort. The symbol "-" indicates no missing data or variable not collected in that cohort.

Table S2. Association between nut consumption and incident dementia: results from Model 1 and Model 2

Variables	Nut Consumption			
	**0 g/Day**	**0.1–5.0 g/Day**	**>5.0 g/Day**	***p* Value for Trend**
HRS				
Cases/Person-years	91/4480	304/24,216	137/16,000	
Nut consumption, median (Q1, Q3)	0	1.42 (0.95, 2.84)	14.51 (7.83, 26.80)	
Model 1, HR (95% CI) *	1 (Reference)	0.76 (0.60, 0.96)	0.60 (0.46, 0.79)	0.003
Model 2, HR (95% CI) †	1 (Reference)	0.80 (0.63, 1.02)	0.60 (0.45, 0.79)	0.001
FOS				
Cases/ Person-years	89/10,643	132/23,577	41/6943	
Nut consumption, median (Q1, Q3)	0	1.89 (0.95, 2.61)	8.75 (6.08, 12.15)	
Model 1, HR (95% CI) *	1 (Reference)	0.85 (0.64, 1.12)	0.82 (0.56, 1.19)	0.382
Model 2, HR (95% CI) †	1 (Reference)	0.84 (0.64, 1.12)	0.82 (0.55, 1.22)	0.428
WHII				
Cases/ Person-years	74/27,956	81/54,064	43/23,035	
Nut consumption, median (Q1, Q3)	0	2.02 (1.35, 2.70)	10.10 (7.09, 14.20)	
Model 1, HR (95% CI) *	1 (Reference)	0.73 (0.53, 1.01)	0.94 (0.64, 1.38)	0.837
Model 2, HR (95% CI) †	1 (Reference)	0.72 (0.52, 0.99)	0.87 (0.59, 1.28)	0.856
Pooled‡				
Model 1, HR (95% CI) *	1 (Reference)	0.78 (0.67, 0.91)	0.75 (0.57, 0.99)	0.011
Model 1, I^2 (%)		0.0	50.6	0.0
Model 1, p_{hetero}		0.751	0.132	0.576
Model 2, HR (95% CI) †	1 (Reference)	0.79 (0.69, 0.93)	0.73 (0.57, 0.94)	0.008
Model 2, I^2 (%)		0.0	31.8	0.3
Model 2, p_{hetero}		0.774	0.231	0.367

[*] Model 1 was adjusted for age, age square, sex, race, marital status, education level, income level, BMI, smoking status, and physical activity.

[†] Model 2 was adjusted for variables in Model 1 plus hypertension, heart disease, stroke, depressive symptoms, and total energy intake.

[‡] Study estimates from three cohorts were pooled using a random-effects model.

Table S3. Association between recommended nut consumption and incident dementia in the HRS[*]

Variables	<5 ounces/Week	≥5 ounces/Week	*p* Value for Trend
Cases/Person-years	481/38,430	51/6266	
Nut consumption, median (Q1, Q3)	2.36 (0.95, 5.00)	30.70 (24.80, 45.00)	
Model 1, HR (95% CI) [†]	1 (Reference)	0.73 (0.55, 0.98)	0.038
Model 2, HR (95% CI) [‡]	1 (Reference)	0.68 (0.50, 0.91)	0.011
Model 3, HR (95% CI) [§]	1 (Reference)	0.70 (0.51, 0.94)	0.019

[*] Recommended nut intake was defined as ≥5 ounces/week based on the Dietary Guidelines for Americans 2020–2025.

[†] Model 1 was adjusted for age, age square, sex, race, marital status, education level, income level, BMI, smoking status, and physical activity.

[‡] Model 2 was adjusted for variables in Model 1 plus hypertension, heart disease, stroke, depressive symptoms, and total energy intake.

[§] Model 3 was adjusted for variables in Model 2 plus the modified MIND diet score.

Table S4. Subgroup analyses of nut consumption (>5.0 g/day vs. 0 g/day) and incident dementia in the three cohorts

Variables	HRS			FOS			WHII		
	Cases/Total	HR (95% CI)	*p* for Interaction	Cases/Total	HR (95% CI)	*p* for Interaction	Cases/Total	HR (95% CI)	*p* for Interaction
Age			0.549			0.375			0.129
<65 years	135/3036	0.67 (0.39, 1.16)		46/1977	1.36 (0.55, 3.34)		52/5613	1.29 (0.58, 2.84)	
≥ 65 years	397/3080	0.56 (0.40, 0.78)		216/1030	0.79 (0.50, 1.24)		146/2613	0.76 (0.48, 1.22)	
Sex			0.294			0.748			0.532
Male	221/2491	0.76 (0.49, 1.19)		106/1368	0.65 (0.35, 1.21)		124/5672	0.77 (0.48, 1.22)	
Female	311/3625	0.51 (0.35, 0.75)		156/1639	0.96 (0.55, 1.68)		74/2544	1.03 (0.50, 2.13)	
Education level			0.394			0.689			0.984
High school or below	365/2941	0.62 (0.45, 0.86)		124/1152	0.85 (0.47, 1.53)		64/2466	0.85 (0.41, 1.77)	
College or above	167/3175	0.68 (0.38, 1.23)		138/1855	0.87 (0.49, 1.53)		134/5760	0.84 (0.53, 1.35)	
Income level			0.388			0.452			0.331
Low	245/1580	0.69 (0.46, 1.03)		137/1248	0.71 (0.39, 1.31)		125/3935	0.79 (0.49, 1.27)	
Medium/high	287/4536	0.51 (0.34, 0.76)		125/1759	0.95 (0.55, 1.64)		73/4291	1.12 (0.55, 2.30)	
BMI			0.961			0.323			0.580
<25.0 kg/m^2	161/1595	0.47 (0.27, 0.82)		84/874	0.73 (0.34, 1.53)		83/3189	0.82 (0.44, 1.53)	
≥25.0 kg/m^2	371/4521	0.67 (0.48, 0.93)		178/2133	1.01 (0.62, 1.65)		115/5037	0.89 (0.54, 1.49)	
Current smoking status			0.584			0.938			0.486
Yes	60/719	0.83 (0.37, 1.84)		24/382	0.73 (0.14, 3.92)		20/866	1.49 (0.38, 5.90)	

No	472/5397	0.59 (0.44, 0.80)		238/2625	0.88 (0.58, 1.34)		178/7360	0.82 (0.54, 1.24)	
Physical activity			0.408			0.827			0.465
Low	433/4356	0.60 (0.44, 0.82)		173/1778	0.97 (0.58, 1.61)		163/6870	0.93 (0.60, 1.44)	
High	99/1760	0.83 (0.37, 1.86)		89/1229	0.83 (0.42, 1.63)		35/1356	0.66 (0.26, 1.68)	

Cox proportional hazards model adjusted for age, age square, sex, race, marital status, education level, income level, BMI, smoking status, physical activity, hypertension, diabetes, heart disease, stroke, depressive symptoms (yes or no), total energy intake, and the modified MIND diet score.

Table S5. Association between nut consumption and incident dementia in sensitivity analyses [*]

Variables	HRS		FOS		WHII		Pooled [†]		
	Cases/ Person-years	HR (95% CI)	Cases/ Person-years	HR (95% CI)	Cases/ Person-years	HR (95% CI)	HR (95% CI)	I^2 (%)	p_{hetero}
Excluding participants with missing covariates									
0 g/Day	86/4274	1 (Reference)	24/4138	1 (Reference)	32/12,850	1 (Reference)	1 (Reference)		
0.1–5.0 g/Day	282/23,298	0.80 (0.63, 1.02)	36/9920	0.65 (0.38, 1.12)	40/32,487	0.59 (0.37, 0.95)	0.74 (0.60, 0.90)	0.0	0.470
>5.0 g/Day	132/15,318	0.64 (0.48, 0.86)	23/2932	1.44 (0.75, 2.79)	24/14,990	0.74 (0.42, 1.29)	0.82 (0.52, 1.28)	59.1	0.087
p Value for Trend		0.011		0.051		0.723	0.792	67.4	0.047
Excluding participants with stroke at baseline									
0 g/Day	75/4104	1 (Reference)	86/10,503	1 (Reference)	69/27,052	1 (Reference)	1 (Reference)		
0.1–5.0 g/Day	268/22,888	0.85 (0.65, 1.10)	130/23,328	0.85 (0.64, 1.13)	76/52,456	0.73 (0.51, 1.02)	0.82 (0.69, 0.97)	0.0	0.754
>5.0 g/Day	117/15,314	0.59 (0.43, 0.80)	36/6803	0.81 (0.54, 1.23)	36/22,435	0.83 (0.55, 1.25)	0.71 (0.56, 0.90)	13.4	0.315
p Value for Trend		<0.001		0.414		0.660	0.004	0.0	0.662
Excluding participants developing dementia in the first 5 years of follow-up									
0 g/Day	73/4088	1 (Reference)	72/10,425	1 (Reference)	69/27,052	1 (Reference)	1 (Reference)		
0.1–5.0 g/Day	254/22,572	0.83 (0.64, 1.09)	119/23,334	0.93 (0.69, 1.27)	76/52,456	0.73 (0.53, 1.02)	0.83 (0.70, 0.99)	0.0	0.570
>5.0 g/Day	111/15,168	0.60 (0.44, 0.82)	40/6895	1.05 (0.69, 1.59)	36/22,435	0.83 (0.55, 1.25)	0.79 (0.56, 1.10)	57.4	0.096
p Value for Trend		0.001		0.730		0.660	0.107	31.5	0.232
Adjusting for other food groups									
0 g/Day	91/4480	1 (Reference)	89/10,643	1 (Reference)	74/27,956	1 (Reference)	1 (Reference)		
0.1–5.0 g/Day	304/24,216	0.88 (0.69, 1.12)	132/23,577	0.91 (0.68, 1.22)	81/54,064	0.73 (0.53, 1.03)	0.85 (0.72, 1.00)	0.0	0.579
>5.0 g/Day	137/16,000	0.68 (0.51, 0.91)	41/6943	0.95 (0.63, 1.45)	43/23,035	0.89 (0.59, 1.33)	0.80 (0.64, 1.00)	5.4	0.348
p Value for Trend		0.008		0.910		0.971	0.021	0.0	0.524
Adjusting for current drinking status									

0 g/Day	91/4480	1 (Reference)	89/10,643	1 (Reference)	74/27,956	1 (Reference)	1 (Reference)		
0.1–5.0 g/Day	304/24,216	0.81 (0.64, 1.03)	132/23,577	0.86 (0.65, 1.14)	81/54,064	0.72 (0.52, 0.99)	0.80 (0.69, 0.94)	0.0	0.713
>5.0 g/Day	137/16,000	0.62 (0.47, 0.82)	41/6943	0.88 (0.59, 1.31)	43/23,035	0.86 (0.58, 1.28)	0.75 (0.58, 0.96)	29.2	0.244
p Value for Trend		0.002		0.646		0.849	0.016	0.0	0.627

[*] Cox proportional hazards model adjusted for age, age square, sex, race, marital status, education level, income level, BMI, smoking status, physical activity, hypertension, diabetes, heart disease, stroke, depressive symptoms, total energy intake, and the modified MIND diet score.

[†] Study estimates from three cohorts were pooled using a random-effects model.
